# Supplementary material for: Viro3D: a comprehensive database of virus protein structure predictions
Source: Mol Syst Biol. 2025 Sep 16;21(11):9. doi: 10.1038/s44320-025-00147-9 (PMC12583693; doi:10.1038/s44320-025-00147-9)
Supplement: Supplementary file 1 — Appendix [file 44320_2025_147_MOESM1_ESM.pdf]

Appendix for: “Viro3D: a comprehensive database of virus protein structure predictions”, Litvin et al. 2025

Table of contents

Page 2: **Appendix Figure S1**. Comparison of Viro3D with alternative repositories of viral protein structure predictions.

Page 3: **Appendix Figure S2.** Homogeneity of structural clusters.

Page 4: **Appendix Figure S3.** Mapping the distribution of hallmark viral proteins across the human and animal virosphere.

Page 5: **Appendix Figure S4.** Propagated protein annotations by viral realm.

Page 6: **Appendix Figure S5.** Genomic positions of singleton (unique) and non-singleton protein-coding genes by viral realm.

Page 7: **Appendix Figure S6.** Grouping viruses by structural similarity recapitulates taxonomy.

Page 8: **Appendix Figure S7.** Structure-informed phylogenetic reconstructions of class-I fusion glycoproteins.

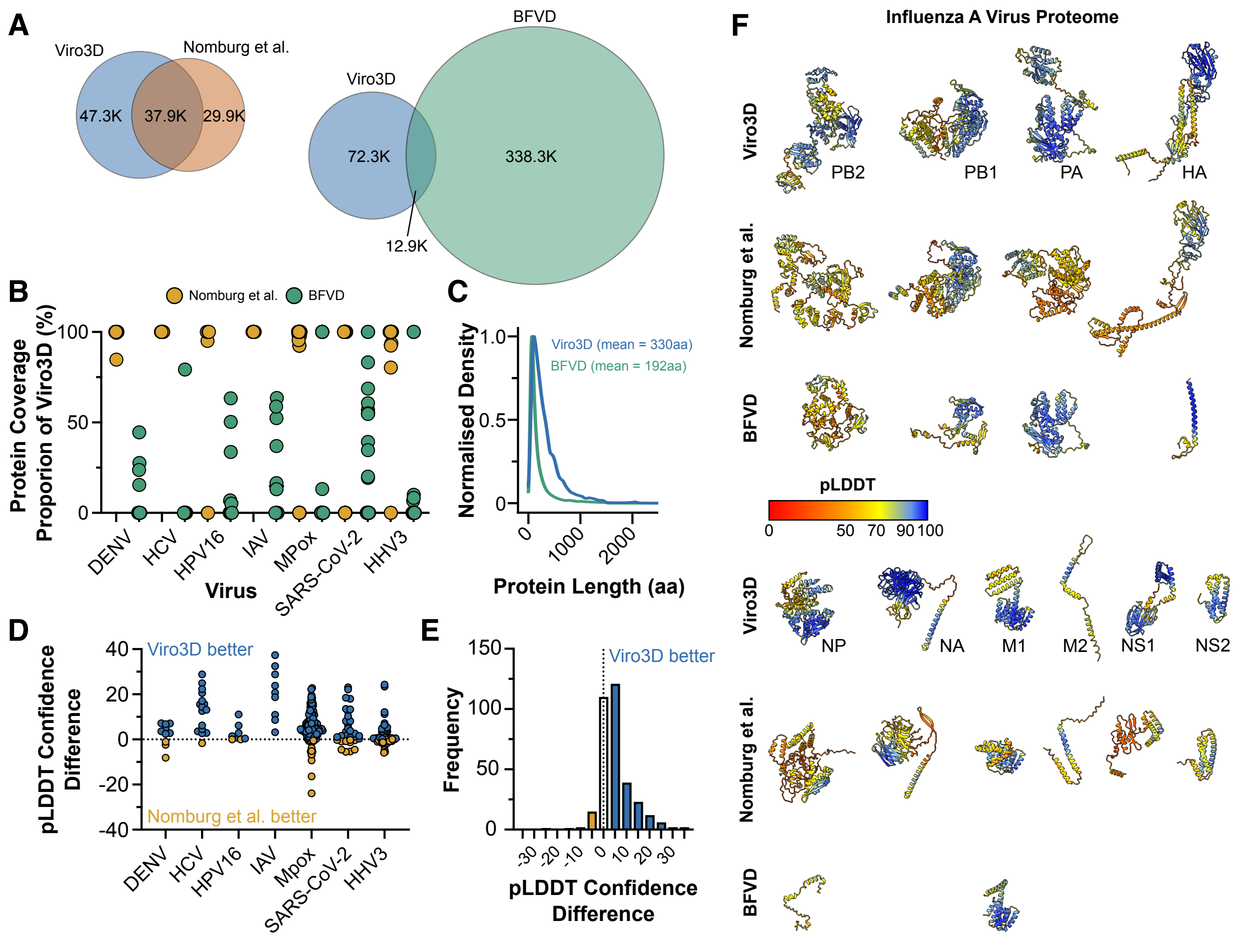

Appendix Figure S1. Comparison of Viro3D with alternative repositories of viral protein structure predictions. Viro3D was systematically compared to the Nomburg et al. dataset and the Big Fantastic Virus Database (BFVD). **A.** Foldseek was used to find matching protein structures with the following threshold parameters: e-value lower than 1e-5, sequence identity ≥95% and query coverage ≥95%. Venn diagrams demonstrate overlap in datasets. We also compared proteome level structure sets from each database for example human pathogens: dengue virus (DENV), hepatitis C virus genotype 1a (HCV), human papilloma virus 16 (HPV16), influenza A virus PR8 (IAV), Mpox, severe acute respiratory syndrome coronavirus-2 (SARS-CoV-2) and human alphaherpes virus 3 (HHV3). **B.** Each dot displays the coverage of individual proteins in the alternative datasets (as a proportion of the Viro3D model). **C.** Distribution of protein lengths (in amino acid residues) for all entries in Viro3D and BFVD. **D.** Proteome level comparisons, as in B, of structure prediction confidence (pLDDT) in matching models from Viro3D and Nomburg et al. Data is expressed as difference in pLDDT scores, with positive values indicating a higher score in the Viro3D model. **E.** Histogram of pLDDT difference for the cumulative set of structures shown in D (n= 334 models). **F.** Predicted Viro3D structures for influenza A virus PR8 proteome alongside the corresponding structures from Nomburg et al. and BFVD. Ribbon diagrams are colour-coded by pLDDT confidence as denoted in the key.

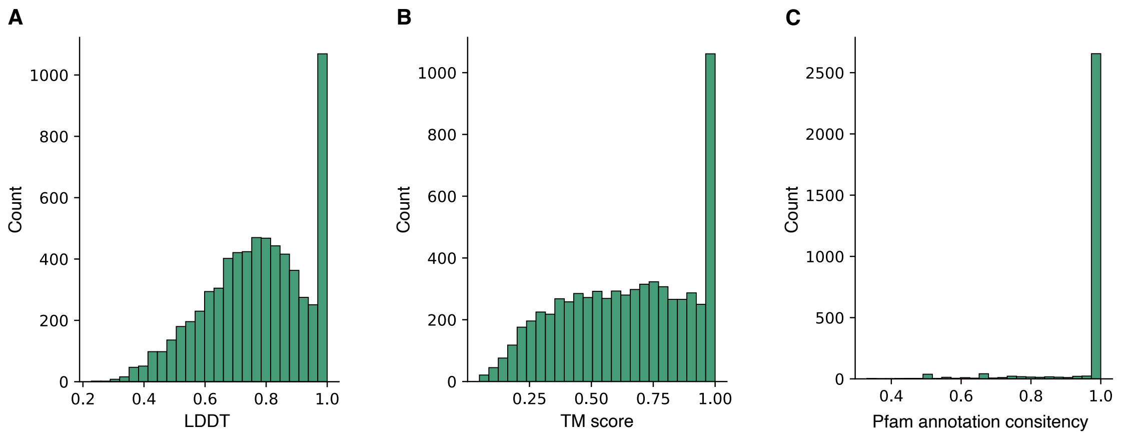

Appendix Figure S2. Homogeneity of structural clusters. A. Distribution of average LDDT scores across all 6,797 non-singleton structural clusters. LDDT provides a measure of local structural similarity, with identical structures giving a value of 1, the median LDDT score is 0.78. **B.** Distribution of average TM scores across all non-singleton structural clusters. TM score gives a measure of global structural similarity, again identical structures give a value of 1, the median TM score is 0.66. **C.** Distribution of Pfam function annotation consistency across 2,963 structural clusters that have at least two members with Pfam annotation. We would expect proteins clustered by structure to have the same function, 95% of annotated clusters have Pfam consistency of 100%.

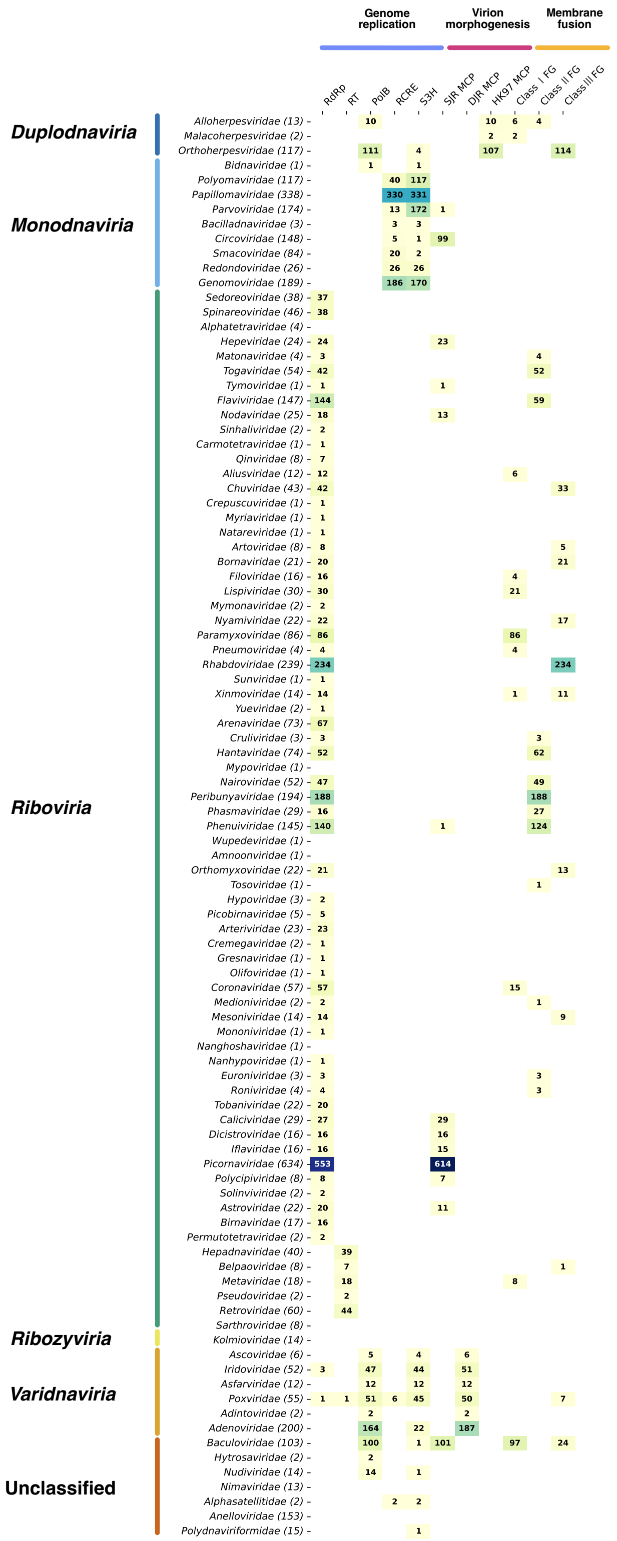

Appendix Figure S3. Mapping the distribution of hallmark viral proteins across the human and animal virosphere. Number of viruses with structural homologs to hallmark viral proteins, organised by virus family. Structural homologs were found using a Foldseek search against the structure similarity network (Fig. 2) using individual experimental protein structures as references: RNA dependent RNA polymerase (PDB ID: 4R0E), reverse transcriptase (1HMV), DNA polymerase B (2PY5), rolling-circle replication endonuclease (8H56), superfamily 3 helicase (5A9K), single jelly roll capsid protein (8E8R), double jelly roll capsid protein (6B1T), HK97 capsid protein (6LGL), class-I fusion glycoprotein (6APB), class-II fusion glycoprotein (6ZQI), class-III fusion glycoprotein (7KDP).

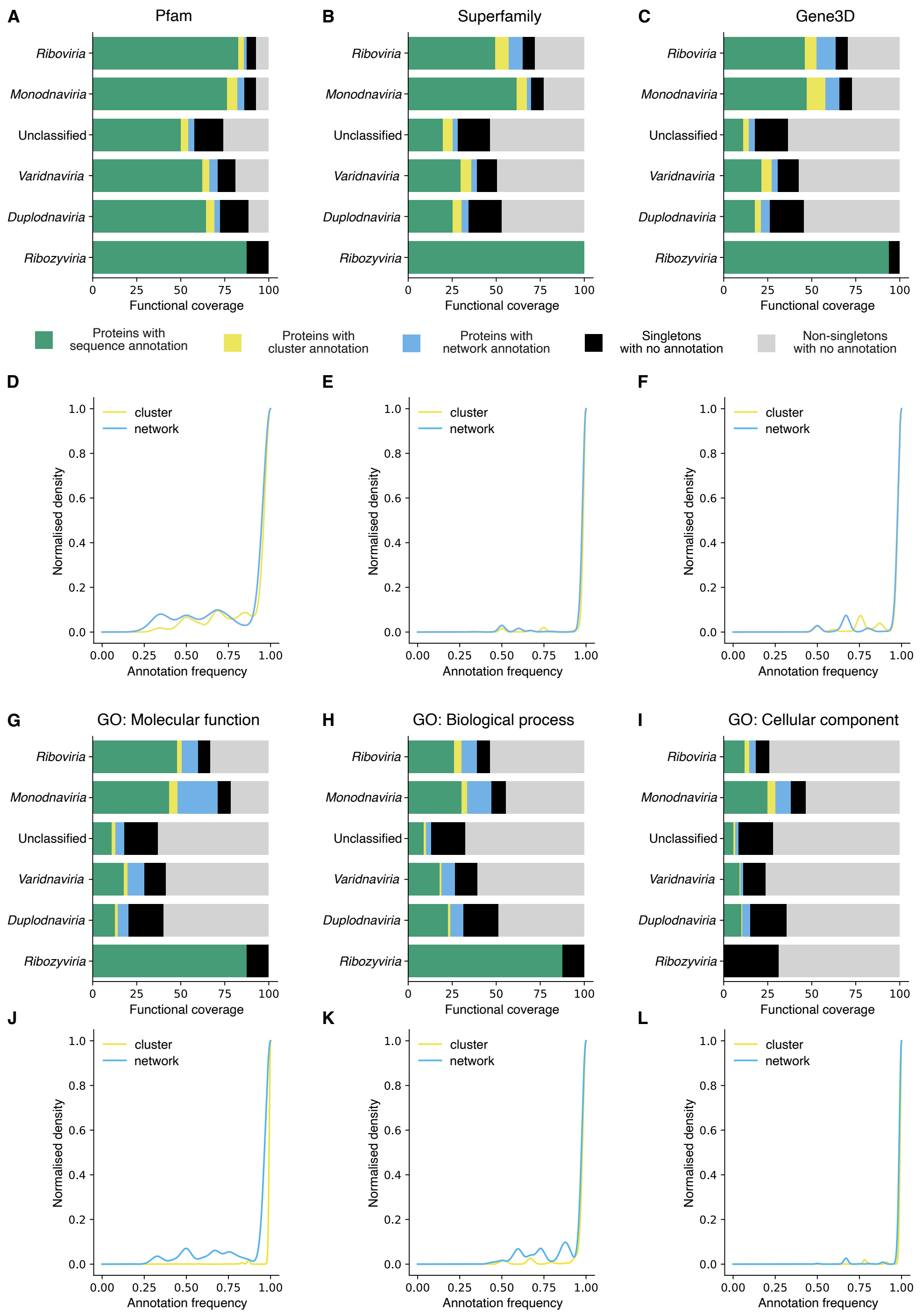

Appendix Figure S4. Propagated protein annotations by viral realm. A-C. and **G-I.** Percentage of protein records in each viral realm that possess functional annotation based on InterProScan (green), structural cluster expansion (yellow), structural network expansion (light blue). Percentage of protein records that do not have a functional annotation are black (if they belong to singleton clusters) or light grey (if they belong to non-singleton cluster). **D-F.** and **J-L.** Distribution of the frequencies of propagated annotations. Functional annotations propagated using structural clusters are in yellow, annotations propagated using structural network are in light blue.

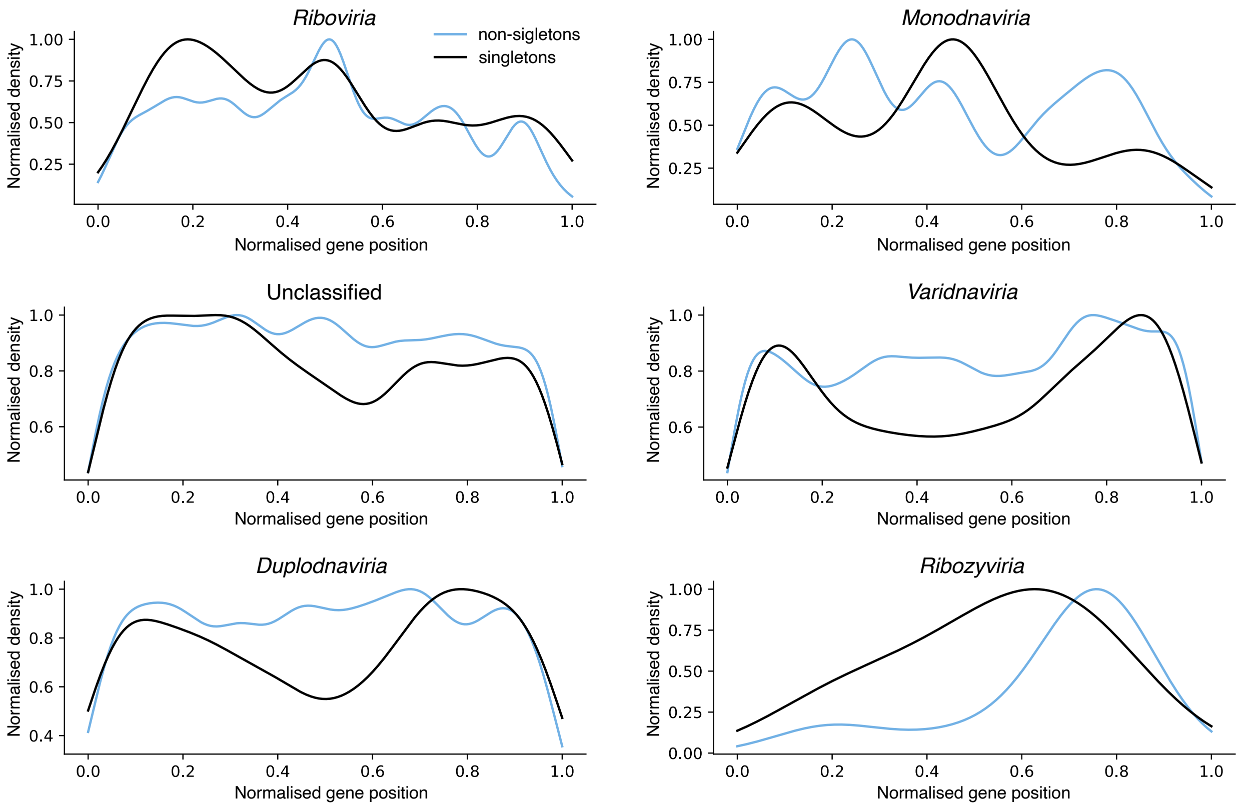

Appendix Figure S5. Genomic positions of singleton (unique) and non-singleton protein-coding genes by viral realm. Proteins that belong to singleton clusters are in black, protein that belong to non-singleton clusters are in light blue.

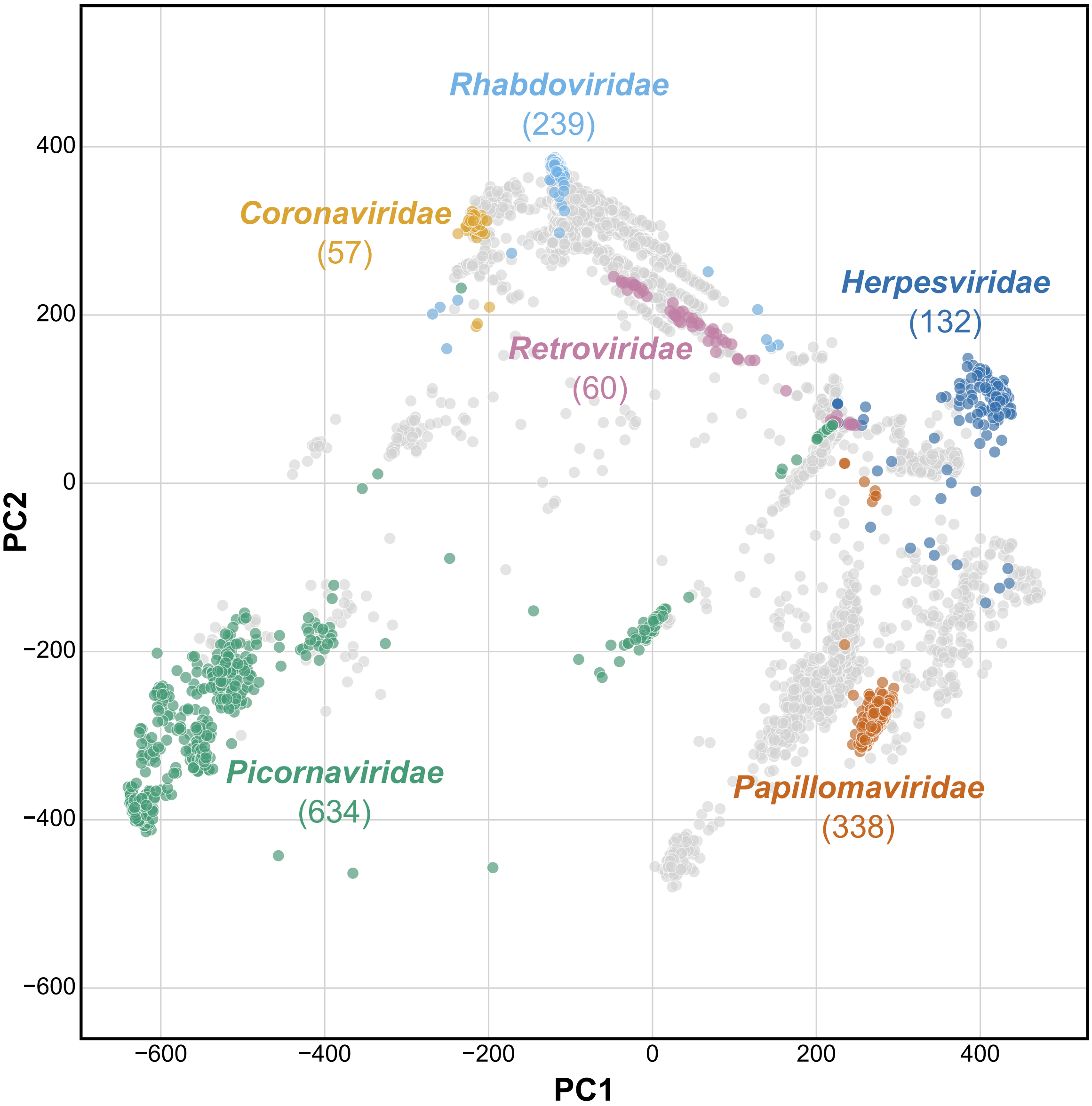

Appendix Figure S6. Grouping viruses by structural similarity recapitulates taxonomy. Structure-informed map of the human and animal virosphere, each data point represents a virus in the Viro3D database (see Methods). Coloured data points show example viral families, which mainly produce coherent clusters within the structure-similarity map. Values in parentheses indicate the number of viruses within each family.

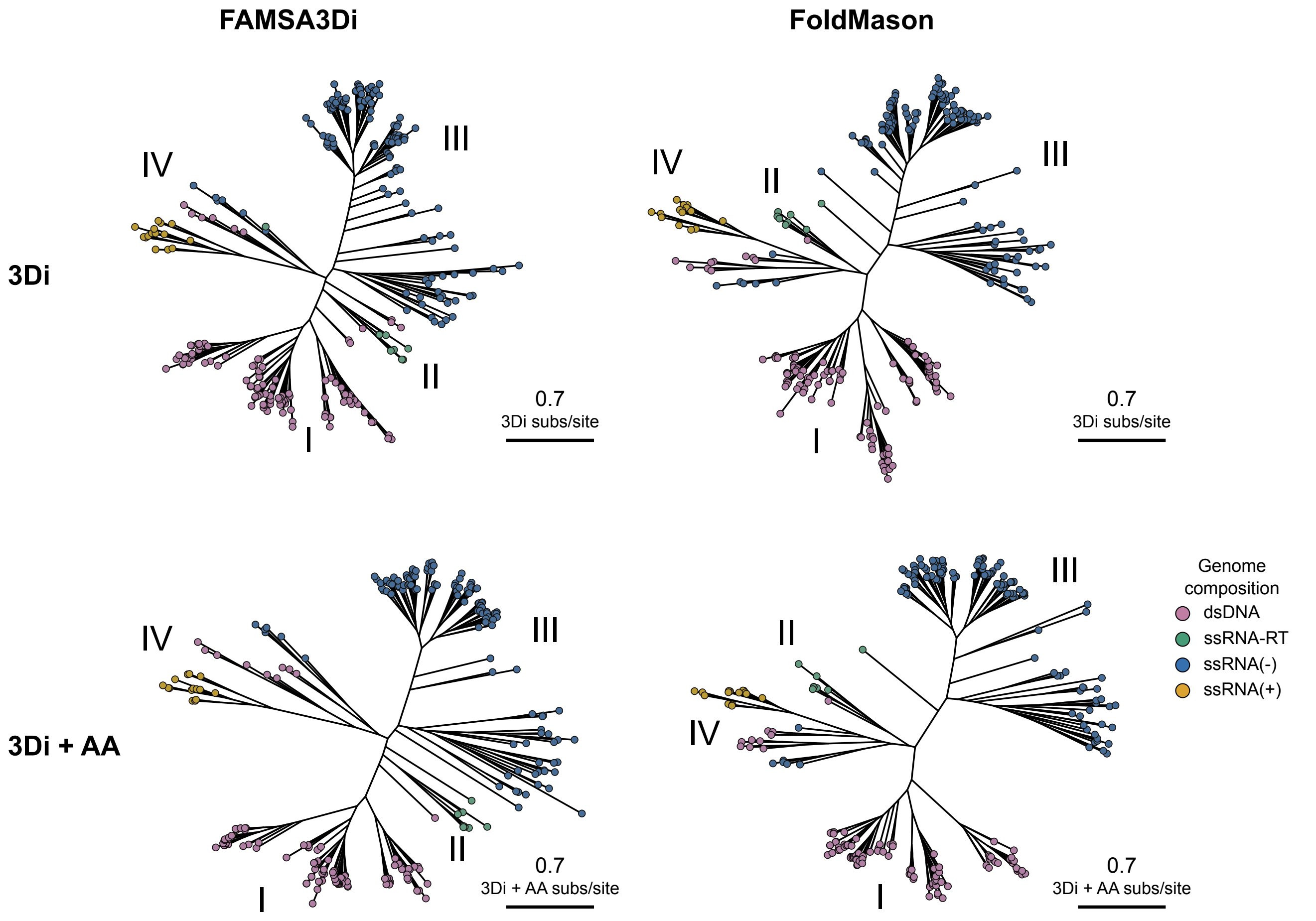

Appendix Figure S7. Structure-informed phylogenetic reconstructions of class-I fusion glycoproteins. Phylogenies based on FAMSA3di (left) and FoldMason (right) alignments using only the 3Di alignment, and combined 3Di and amino acid alignments (top to bottom). Scale bars on the right of each tree represent 3Di substitutions per site for 3Di only inferences and 3Di and amino acid substitutions per site for 3Di + AA inferences. Tips are coloured by the virus genome composition, and the major clades are labelled with roman numerals as in Fig. 3C.
