# Supplementary material for: Viro3D: a comprehensive database of virus protein structure predictions
Source: Mol Syst Biol. 2025 Sep 16;21(11):9. doi: 10.1038/s44320-025-00147-9 (PMC12583693; doi:10.1038/s44320-025-00147-9)
Supplement: Supplementary file 12 — Expanded View Figures [file 44320_2025_147_MOESM12_ESM.pdf]

Expanded View Figures

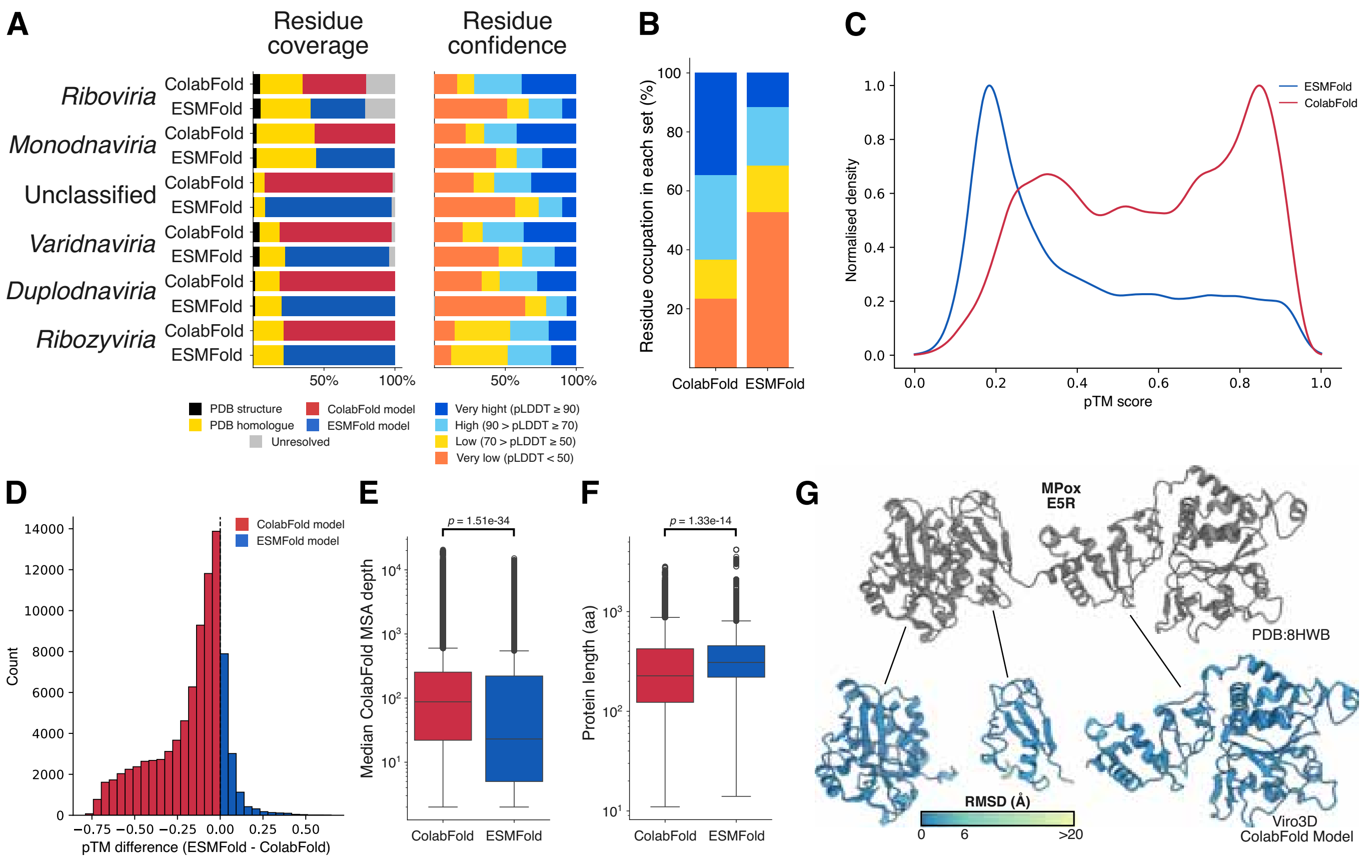

Figure EV1. Expanding structural coverage of the human and animal virosphere.

(**A**) On the left, percentage of residues covered by ColabFold (red) and ESMFold (blue) models in contrast to percentage of residues covered by PDB structures (sequence identity ≥ 95%, black) and PDB homologs (sequence identity ≥30%, yellow) for each viral realm. On the right, confidence of residues modeled by ColabFold and ESMFold based on pLDDT score per viral realm. (**B**) Percentage of ColabFold and ESMFold residues with very high (dark blue), high (light blue), low (yellow) and very low (orange) pLDDT score. (**C**) Normalized distribution of pTM scores for ColabFold (red, median pTM score of 0.59) and ESMFold (blue, median pTM score of 0.31) models. (**D**) Distribution of differences in pTM scores between ColabFold and ESMFold models for each protein record where both models are available. Positive values indicate that ESMFold model is better (blue bars), negative value – ColabFold model is better (red bars). 15.74% of protein records have a higher pTM score with ESMFold prediction, 848 of these records have difference in pTM score greater than 0.2. (**E**) Distributions of median ColabFold MSA depth for records where ColabFold model has a higher pLDDT score (red box) and ESMFold models has a higher pLDDT score (blue box). The difference between MSA depth is significant based on the *t* test results (t-statistic: -12.26; *p*-value: 1.51e-34). (**F**) Distributions of protein length for records where ColabFold model has a higher pLDDT score (red box) and ESMFold models has a higher pLDDT score (blue box). The difference between protein length is significant based on the *t* test results (t-statistic: -7.70; *p*-value: 1.33e-14). In both (**E**) and (**F**), the bounds of boxes represent the interquartile range (IQR, 25th–75th percentile), with the center line showing the median (50th percentile). Whiskers extend to the minima and maxima within 1.5 × IQR. Individual data points outside the whiskers are outliers; ColabFold (n = 77,071), ESMFold (n = 7769). (**G**) Mpox E5R protein, as shown in Fig. 1G, but with the three constituent individual domains color-coded by RMSD (Å) after their respective rigid-body alignment with the experimental structure (gray). This indicates that whilst their relative positions may diverge from the experimental structure (Fig. 1G), the individual domains are accurately predicted.

 © The Author(s)

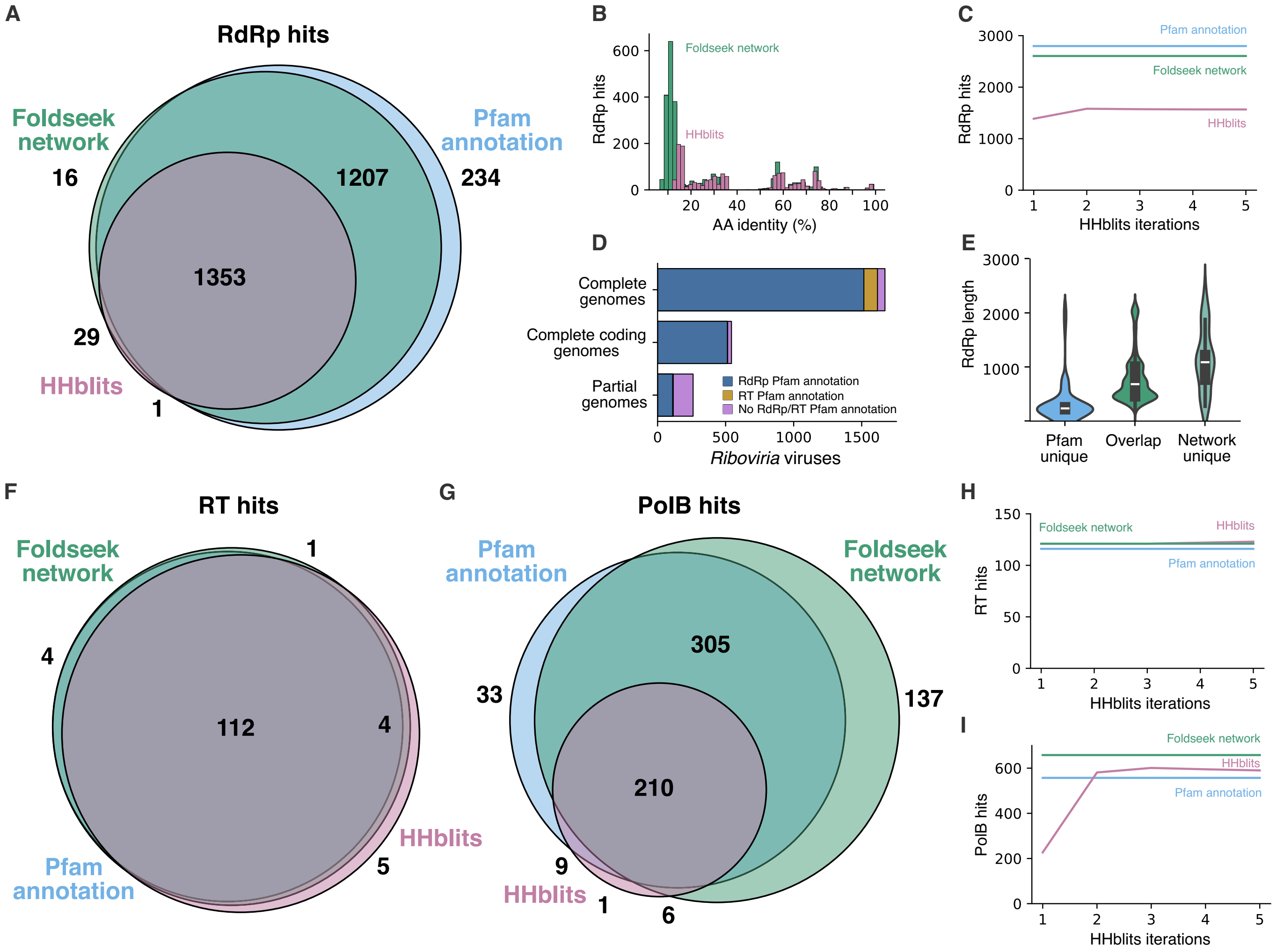

Figure EV2. Comparison of structure and sequence-based mapping of viral polymerases.

(**A**) Venn diagram demonstrating the overlap between the number of proteins with RdRp Pfam annotation (in light blue color) and RdRp hits found using one iteration of HHblits search (in pink) or Foldseek search against structural network (in green). (**B**) Distribution of pairwise amino acid identities between RdRp probe (PDB ID: 4R0E) and Viro3D entries identified through one iteration using HHblits (pink) or Foldseek network search (green). (**C**) Number of RdRp hits found after five HHblits search iterations in comparison to the number of records with RdRp Pfam annotation and records found using Foldseek search against structural network. (**D**) Number of viruses in the *Riboviria* that possess proteins with RdRp or RT Pfam annotation split into groups based on sequence entry genome completeness. (**E**) Distribution of protein length for RdRp records unique to Pfam annotation, Foldseek network search or present in both. The white dot in the center of the violin represents the median of the data (50th percentile). The thick bar shows the interquartile range (IQR, 25th–75th percentile). The thin line extending from the box shows the range of the data within 1.5 × IQR. The tips of the violin represent the minimum and maximum values in the data, as far as the density estimate extends. Pfam unique ($n = 238$), overlap ($n = 2560$), network unique ($n = 45$). (**F**) Venn diagram highlighting the overlap between the number of proteins with RT Pfam annotation and RT hits found using one iteration of HHblits search or Foldseek search against the structural network. (**G**) Venn diagram showing the overlap between the number of proteins with PolB Pfam annotation and PolB hits found using one iteration of HHblits search or Foldseek search against structural network. (**H**) Number of RT hits found after five HHblits search iterations in comparison to the number of records with RT Pfam annotation and records found using Foldseek search against structural network. (**I**) Number of PolB hits found after five HHblits search iterations in comparison to the number of records with PolB Pfam annotation and records found using Foldseek search against structural network.

© The Author(s)

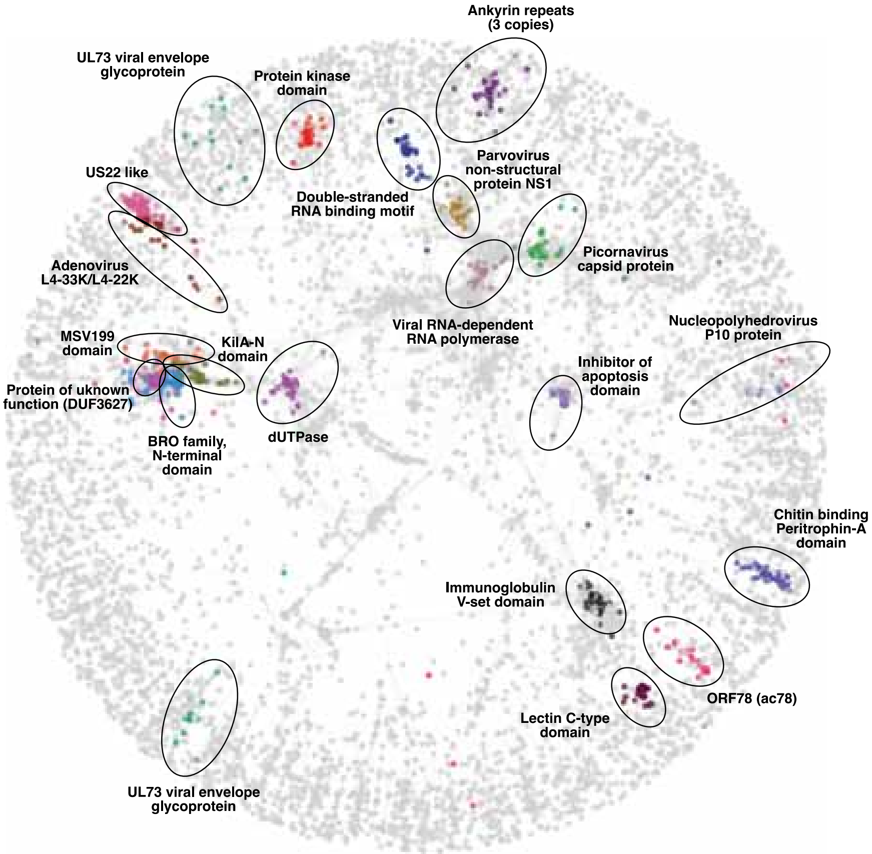

Figure EV3. Functionally annotated structure similarity network of viral proteins.

Each node represents a cluster. Edges connect clusters that share structural similarity. Clusters that possess the twenty most frequent Pfam annotations are highlighted in different colors.

 © The Author(s)

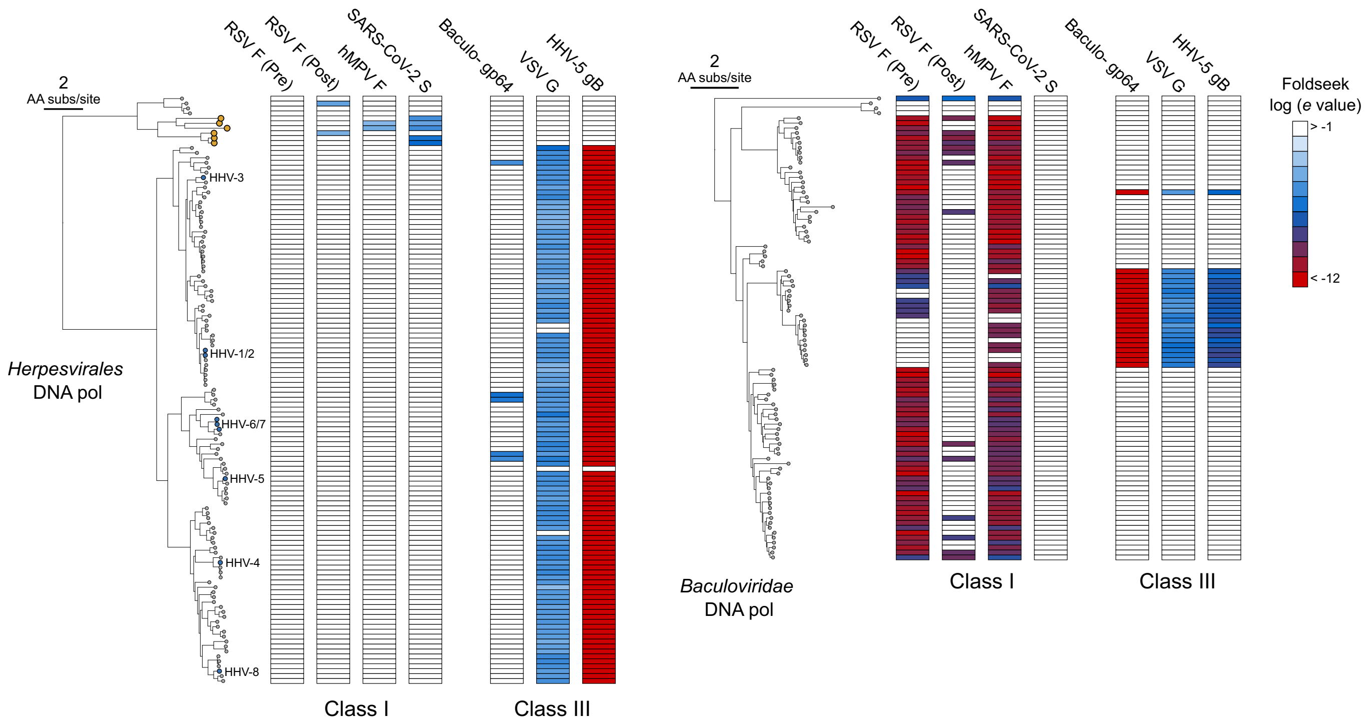

Figure EV4. Foldseek homology for class-I and class-III fusion proteins in the *Herpesvirales* and *Baculoviridae*.

Whole proteomes for all species within either taxonomic group were surveyed, using Foldseek, for structural homology against class-I and class-III fusion proteins. Foldseek structural homology scores were mapped against the underlying DNA polymerase amino acid sequence phylogeny to reveal the distribution of fusion mechanisms. Presented phylogenies are midpoint rooted. Heatmaps display Foldseek log transformed *e*-values (as indicated in the key) for the stated references, including RSV F protein in both pre- and post-fusion states (see Methods for details). For orientation in the *Herpesvirales* phylogeny, human herpesviruses have tips colored blue with species labels, whereas the aquatic herpesviruses identified in our structural cluster search (Fig. 3B) are colored yellow. Scale bar represents amino acid substitutions per site. hMPV human metapneumovirus, VSV vesicular stomatitis virus, HHV-5 human herpesvirus-5.

© The Author(s)
